# Preparation, Separation, and Identification of Low-Bitter ACE-Inhibitory Peptides from Sesame (*Sesamum indicum* L.) Protein

**DOI:** 10.3390/foods15020279

**Published:** 2026-01-12

**Authors:** Xin Lu, Cong Jia, Lixia Zhang, Xiaojing Sun, Guohui Song, Qiang Sun, Jinian Huang

**Affiliations:** 1 Research Center for Agricultural Products Processing, Henan Academy of Agricultural Sciences, 116 Park Road, Zhengzhou 450002, China; xinlu1981@foxmail.com (X.L.); 18300697186@163.com (X.S.); sigehe@126.com (G.S.); qiangsunxy@126.com (Q.S.); jinianhuang71@gmail.com (J.H.); 2 School of Food Science and Engineering, Zhengzhou University of Science and Technology, 1 College Road, Zhengzhou 450064, China; 18737181615@163.com

**Keywords:** molecular docking, multi-enzyme hydrolysis, molecular dynamics stimulation, sesame protein, bitter taste receptor

## Abstract

To prepare and characterize low-bitter angiotensin-converting enzyme (ACE)-inhibitory peptides from sesame protein, a triple-enzyme hydrolysis system was optimized using mixture design and response surface methodology. The resulting hydrolysate was separated by ultrafiltration and medium-pressure chromatography, followed by identification through nano-liquid chromatography–electrospray ionization-tandem mass spectrometry. Finally, the mechanism of typical low-bitter ACE-inhibitory peptides was elucidated by molecular docking and molecular dynamics simulation. Results showed that the optimal enzyme activity ratio of 1:0.94:1.07 for Alcalase, trypsin, and Flavourzyme, combined with optimized hydrolysis conditions (E/S ratio of 126,793.03 nkat/g, pH 8.40, 4.82 h hydrolysis time, and 45 °C), resulted in a peptide yield of 93.19 ± 0.14%, ACE-inhibitory rate of 95.92 ± 0.23%, and bitter value of 3.15 ± 0.09. APQLGR and APWLR exhibited high ACE-inhibitory activity and minimal bitterness among the seventeen identified peptides. Although both peptides bound to the S1 pocket and Zn^2+^ catalytic site of ACE, APWLR exhibited an additional interaction with the S2 pocket. Both peptides were predicted to antagonize the bitter taste receptor T2R14 by forming stable complexes with key residues, but two complexes exhibited distinct mechanisms of stabilization. This work demonstrates a method for producing dual-functional peptides from sesame protein, paving the way for their application in functional foods.

## 1. Introduction

Hypertension is a significant risk factor for cardiovascular disease and stroke [1]. With over 1 billion people affected worldwide, it has become a critical global public health issue [2]. Angiotensin I-converting enzyme (ACE, EC 3.4.15.1) inhibitors targeting the renin-angiotensin system (RAS) have been considered key medications for hypertension [1]. Owing to their low adverse effects, ACE-inhibitory peptides derived from various edible proteins are increasingly recognized as potential alternatives to synthetic antihypertensive drugs [3].

Sesame (*Sesamum indicum* L.) is a worldwide oilseed (Figure S1), with a global production of 6.80 million tons in 2020. Sesame seed is primarily processed for edible oil, generating 1.53 million tons of sesame meal annually as a by-product [4]. Sesame meal, with a protein content of about 50% [5], is recognized as a promising protein source. To utilize sesame meal, previous studies have reported that enzymatic hydrolysis of sesame protein could produce bioactive peptides, including ACE-inhibitory peptides [5,6,7,8].

Similar to other ACE-inhibitory peptides [9], sesame protein hydrolysate with ACE inhibitory activity also tastes bitter [8], which limits their acceptability and has spurred efforts to mitigate the bitterness. Current strategies could be classified into bitterness masking and the reduction of bitter peptides. Bitterness masking involves either adding sweeteners or flavoring agents to interfere with nerve impulse transmission [10], or using antagonists or blockers of bitter taste receptors (T2Rs) [11,12]. Specifically, several peptides have been identified as potential blockers for receptors, such as T2R4, T2R7, T2R14, T2R16, T2R43, and T2R46 [13,14,15]. Conversely, the strategies for removing or reducing bitter peptides include enzymatic modification by exopeptidases [9], isoelectric precipitation, solvent extraction, and adsorption using chromatographic resins or activated carbon [16]. Flavourzyme, a mixture of exo- and endo-peptidases, has been shown to effectively reduce bitter peptides [17,18,19], indicating that low-bitter ACE-inhibitory peptides from sesame protein could be obtained by incorporating Flavourzyme during proteolysis.

The incorporation of Flavourzyme would significantly alter the hydrolysis conditions required to produce low-bitter ACE-inhibitory sesame peptides, distinguishing this method from previous research that utilized simulated gastrointestinal digestion in vitro or dual-enzyme (Alcalase and trypsin) hydrolysis [7,8]. Additionally, the potential blocking effects of these sesame peptides on T2Rs have not yet been investigated. Therefore, this study aims to prepare low-bitter ACE-inhibitory peptides from sesame protein by establishing a triple-enzyme consisting of Alcalase, trypsin, and Flavourzyme via mixture design based on a previous study [8], optimizing its hydrolysate conditions by response surface methodology (RSM), separating and identify the low-bitter ACE-inhibitory peptides, and exploring the interaction mechanisms of promising peptides with ACE inhibitory activity and R2Ts antagonist by molecular docking and molecular dynamics (MD) simulation. This work could facilitate the development of functional foods enriched with ACE-inhibitory peptides to help meet the growing demand for hypertension management.

## 2. Materials and Methods

### 2.1. Materials and Regents

Zhengzhi 13 sesame seed was provided by the Research Center for Sesame of Henan Academy of Agricultural Sciences (Zhengzhou, Henan, China). Alcalase 2.4 L (Protease from *Bacillus licheniformis*, 29.67 × 10^5^ nkat/g), Hippuryl-His-Leu (HHL), lisinopril, and angiotensin I-converting enzyme (ACE, from rabbit lung) were purchased from Sigma–Aldrich (St. Louis, MO, USA). Trypsin (Porcine, 39.17 × 10^5^ nkat/g) was provided by Aladdin Biochemical Technology Co. Ltd. (Shanghai, China). Flavourzyme (Protease from *Aspergillus oryzae*, 32.84 × 10^5^ nkat/g) was obtained from Angel Yeast Co., Ltd. (Yichang, China). Ultrafiltration membranes (UE020, UE010, UE005, UE003, UX001, with molecular weight cut-offs (MWCO) of 20, 10, 5, 3, and 1 kDa, respectively) and Nanofiltration membrane NF1 (MWCO of 150 Da) were purchased from Rising Sun Membrane Technology Co. Ltd. (Beijing, China). Other reagents and chemicals were of analytical grade and supplied by Sinopharm Chemical Reagent Co., Ltd. (Shanghai, China).

### 2.2. Preparation of Sesame Protein

Sesame protein was isolated from white sesame seeds following the method de-scribed by Lu et al. [8]. After washing twice with distilled water, the protein precipitate was lyophilized and stored at 4 °C.

### 2.3. Mixture Design of Triple-Enzyme System for Preparing Low-Bitter ACE-Inhibitory Peptide

According to a previous study [8], a dual-enzyme system of Alcalase and trypsin at a 3:7 enzyme activity ratio was suitable for hydrolyzing sesame protein to produce ACE-inhibitory peptides. Unfortunately, the obtained hydrolysate exhibited a distinctly bitter taste, likely due to the formation of bitter peptides during dual-enzyme hydrolysis. To address this issue, Flavourzyme was introduced into the dual-enzyme system. The mixture design, a special class of response surface design that is qualified for analyzing the proportions of the components or factors through fewer experimental runs [20], was applied. The simplex-lattice model with lattice degree set to 2 for the mixture of the three ingredients Alcalase (A), trypsin (T), Flavourzyme (F), with hydrolysis time (H) as a procedure variable, was designed by Minitab17 (Minitab Ltd., Coventry, UK) to obtain the optimal triple-enzyme composition and analyze the effect of the independent variables. Based on the previous report [8], the temperature, pH, solid–liquid ratio, and E/S were fixed at 45 °C, 8.35, 8% (*w*/*v*), and 103,354 nkat/g, respectively. The detailed settings of hydrolysis time and protease combinations are presented in Table 1.

### 2.4. Optimization of Hydrolysis Conditions by RMS

To further optimize the hydrolysis conditions for preparing the low-bitter ACE-inhibitory peptide from sesame protein using the triple-enzyme system, RSM was applied to evaluate the effects of E/S (X_1_), pH (X_2_), and temperature (X_3_) on peptide yield (Y_1_), ACE inhibitory rate (Y_2_), and bitter value (Y_3_). A Box–Behnken design with 15 experimental runs was constructed by Minitab17 (Table 2).

### 2.5. Fraction of Low-Bitter ACE-Inhibitory Peptide Using Ultrafiltration

Prior to ultrafiltration, the sesame protein hydrolysate underwent desalting using nanofiltration, as described by Lu et al. [8]. The desalination process was repeated three times to obtain desalted sesame protein hydrolysate (DSPH), which was then subjected to ultrafiltration, as previously reported [8]. Sequentially, ultrafiltration through membranes UE020, UE010, UE005, UE003, and UX001 yielded six fractions: DSPH-I (>20 kDa), DSPH-II (10–20 kDa), DSPH-III (5–10 kDa), DSPH-IV (3–5 kDa), DSPH-V (1–3 kDa), and DSPH-VI (<1 kDa). All fractions were lyophilized, stored at −20 °C, and subsequently assayed for their ACE inhibitory activities and bitter value.

### 2.6. Purification by Medium-Pressure Chromatography System

The lyophilized DSPH-VI was dissolved in distilled water (50 mg/mL), then filtered through a 0.2 μm filter, and subsequently purified by an NGC Quest^TM^ 10 Plus chromatography system (Bio-Rad Laboratories, Inc., Hercules, CA, USA) equipped with a C18 preparative column (20 mm × 250 mm I.D., 10 μm, Shimadzu, Kyoto, Japan). The column was eluted by a linear gradient of methanol (50–75%, *v*/*v*) containing 0.1% (*v*/*v*) trifluoroacetic acid (TFA) at a flow rate of 7.5 mL/min for 32 min. The eluent was monitored at 220 nm, and the collected peaks were lyophilized for further measurement of ACE-inhibitory activity and bitterness.

### 2.7. Identification by Nano LC-MS/MS

The peptides in the eluted peak exhibiting the strongest ACE inhibition were identified by a Nano LC-MS/MS system comprising an Easy-nLC 1 200 UHPLC (Thermo Fisher Scientific, Waltham, MA, USA) with an Acclaim PepMap RPLC C18 (1.9 μm, 100 Å, Dr. Maish GmbH, Germany), and a Q Exactive Hybrid Quadrupole–Orbitrap TM mass spectrometer (Thermo Fisher Scientific, Waltham, MA, USA) with an ESI nanospray source. Before LC-MS/MS analysis, samples were reduced by 10 mM Dithiothreitol (DTT) at 56 °C for 1 h, alkylated by 50 mM indole-3-acetic acid (IAA) at room temperature in the dark for 40 min, and lyophilized. The lyophilized sample was dissolved in 0.1% (*v*/*v*) formic acid in water and then filtered through the 0.22 μm filter membrane. In the UHPLC system, the mobile phase consisted of 0.1% (*v*/*v*) formic acid in water (A), and 80% acetonitrile/20% water with 0.1% (*v*/*v*) formic acid (B). The gradient elution was performed as follows: 6–14% B (0–20 min), 14–30% B (20–50 min), and 30–95% B (50–60 min) at 600 nL/min.

The mass spectrometer parameters were as follows: 2.2 kV spray voltage, 270 °C capillary temperature, MS precursor range from 300 m/z to 1400 m/z, and resolution of 70,000 in MS stage; activation type of Higher Energy Collision Dissociation (HCD) and resolution of 17,500 in MS/MS stage. The raw MS files were processed with PEAKS studio 8.5 using de novo peptide sequencing, with a maximum missed cleavages of two, a precursor ion mass tolerance of 20 ppm, and a MS/MS tolerance of 0.02 Da. Only peptides identified with high confidence were retained for subsequent protein identification.

### 2.8. Peptide Synthesis

Peptides were synthesized using the solid-phase synthesis technology by Wuxi Asiapeptide Biotechnology Co., Ltd. (Wuxi, Jiangsu, China). The purity of the synthesized peptide was confirmed to be >95% by HPLC.

### 2.9. Molecular Docking Between ACE and Peptides

The receptor ACE/Lisinopril complex (1O86) was downloaded from the RCSB database (https://www.rcsb.org/structure/1o86 (accessed on 30 September 2024)). Molecular docking between ACE and peptides was performed using Molecular Operating Environment (MOE) software (Version 2022, Chemical Computing Group, Montreal, Canada) according to the procedure described by Lu et al. [8].

### 2.10. Molecular Docking and MD Simulation for T2R14 and Peptides

Among T2Rs, T2R14 shows low ligand selectivity and could be activated by a wide range of bitter compounds [12,21]. Therefore, T2R14 is commonly used to assess whether a compound is bitter. As described by Yu et al. with some modifications [22], the X-ray crystal structure of T2R14 (BitterDB ID: 14) was obtained from the BitterDB Protein Data Bank (https://bitterdb.agri.huji.ac.il (accessed on 5 October 2024)), then prepared by adding hydrogen atoms and removing inactive sites. The identified peptides were processed using ‘Prepare Ligands’ and ‘Minimization’ tools in the small molecule module. The receptor-ligand interaction was analyzed using the CDOCKER method in the Discovery Studio 2019 software (Dassault Systèmes Biovia Corp, Paris, France). Docking parameters included the binding area with coordinates (x, y, z) at (1.21, 2.05, 12.05) with a radius of 17.6 Å, and the pose cluster radius in top hits of 0.5.

MD simulations were performed using GROMACS 2023.3 to evaluate the stability of the peptide–T2R14 complex. The initial coordinates for the complex were obtained from molecular docking. The AMBER14SB and General AMBER Force Field (GAFF) were employed for T2R14 and peptides, respectively [23,24]. The system was prepared using the pdb2gmx module to add hydrogen atoms, solvated in a cubic TIP3P water box, and neutralized with Na^+^/Cl^−^ ions [25]. Energy minimization was performed using the steepest descent algorithm until the maximum force fell below 1000 kJ·mol^−1^·nm^−1^. This was followed by a 100 ps equilibration under an NVT ensemble at 310.15 K, and then another 100 ps equilibration under an NPT ensemble at 1.0 × 10^5^ Pa. Finally, a 100 ns production MD simulation was conducted. During the simulation, the LINCS algorithm was used to constrain all bonds involving hydrogen atoms under the 2 fs integration time step. Electrostatic interactions were calculated using the Particle Mesh Ewald (PME) method with a 1.2 nm cutoff, while van der Waals interactions were truncated at a 1.0 nm cutoff. Trajectory analysis included the calculation of root-mean-square deviation (RMSD), root-mean-square fluctuation (RMSF), radius of gyration (Rg), and intermolecular hydrogen bonds. The binding free energy between peptide ad T2R14 was calculated using the Molecular Mechanics/Generalized Born Surface Area (MM/GBSA) method [26,27].

### 2.11. Determination of Peptide and Protein Concentration

The peptide and protein contents were determined by OPA spectrophotometric assay and Bradford method [8]. The peptide yield was calculated as the percentage of peptide mass to protein mass in the feed. The peptide (protein) mass was calculated by multiplying its concentration by the volume of the corresponding solution.

### 2.12. ACE Inhibitory Activity Assay

Samples were diluted to a peptide concentration of 1 mg/mL for the ACE inhibitory activity assay, with Lisinopril as the positive control. The ACE inhibitory activity assay was determined by the method noted by Lu et al. [8]. The ACE inhibitory rate was calculated as follows,

(1) $$\mathrm{ACE}\;\mathrm{inhibitory}\;\mathrm{rate}\;(\%)=[1-\frac{\left(a-c\right)}{\left(b-d\right)}]\times 100$$

where a, b, c, and d represent the fluorescence intensities of the ACE solution with ACE inhibitors, the ACE solution alone, the ACE inhibitors, and the buffer, respectively. The IC_50_ value was derived from the graph plotting peptide concentration against the ACE inhibitory rate.

### 2.13. Bitterness Testing by Electronic-Tongue (E-Tongue)

Each sample was dissolved in distilled water to a peptide concentration of 10 mg/mL and filtered through a 0.45 μm filter. The Bitterness of the filtered sample (30 mL) was tested using a TS-500Z E-tongue (Intelligent Sensor Technology, Inc., Atsugi, Japan) equipped with six sensors (AAE, CT0, CA0, C00, AE1, GL1). Standard preparation, washing, sample solutions, and testing procedures followed the method described by Zhang et al. [28]. Each sample was tested in triplicate. In each measurement cycle, the reference solution (30 mM potassium chloride and 0.3 mM tartaric acid in distilled water) was measured first, followed by the sample solution [28]. The bitterness value was expressed as a dimensionless taste value calculated by the TS-500Z software (Version 4.1) from the change in membrane potential of the bitterness sensor (C00) relative to the reference electrode [29]. A higher bitterness value indicates stronger bitterness, and the bitterness value of the reference solution was set to 0.

### 2.14. Statistical Analysis

Data were analyzed using Minitab 17 and SAS 9.2 software. Differences were considered significant at *p* < 0.05 and highly significant at *p* < 0.01.

## 3. Results and Discussion

### 3.1. Analysis of Triple-Enzyme for Preparing Low-Bitter ACE-Inhibitory Peptides by Mixture Design

According to the mixture design results (Table 1), dual- or triple-enzyme systems always produced more peptides than single proteases, due to the greater diversity of cleavage sites. Mixed proteases containing trypsin demonstrated stronger ACE-inhibitory activity compared to those without trypsin. This phenomenon was attributed to the presence of Arg (R) or Lys (K) at the C-terminal position of the peptide, generated by trypsin, as these residues enhanced ACE-inhibitory potency [30]. The addition of Flavourzyme reduced the bitterness of SPH dramatically, e.g., the bitter value decreased from 5.98 (Run 1, Alcalase alone) to 5.08 (Run 5, Alcalase–Flavourzyme system). Alcalase is an in-depth endoprotease that cleaves peptide bonds on the C-terminal side of hydrophobic amino acids [31,32], thereby exposing buried hydrophobic residues strongly associated with the peptide bitterness [33], resulting in hydrolysates with higher bitterness. Flavourzyme is a protease preparation enriched in exopeptidases (Leucine aminopeptidase A, Leucine aminopeptidase 2, Dipeptidyl peptidase 4, Dipeptidyl peptidase 5, carboxypeptidase) with additional endoproteases (Neutral protease 1, Neutral protease 2, Alkaline protease 1) and minor α-amylase activity [34,35]. Its predominant exopeptidase activity removes hydrophobic amino acids from peptide termini, effectively debittering the hydrolysate [36]. Notably, the triple-enzyme system composed of Alcalase, trypsin and Flavourzyme proved highly effective in producing low-bitter ACE-inhibitory peptides.

ANOVA results (Table 3) showed that the mathematical models for peptide yield, ACE-inhibitory rate, and bitter value were valid (R^2^ > 95%, *p* < 0.05). The regression equations were qualified to predict the performance of the triple-enzyme system in producing low-bitter ACE-inhibitory peptides from sesame protein.

For peptide yield, the linear factor, binary and ternary interactions without hydrolysis time were significant. Among interactions involving hydrolysis time, only A*H and F*H were significant. The regression equation indicated that the ternary interaction A*T*F exerted the strongest positive effect on peptide yield, consistent with the expectation that combining three proteases provides the widest range of cleavage sites and thus generates more peptides.

For the ACE-inhibitory rate, A*T, A*F, T*F were highly significant factors, individual protease and A*T*F were significant factors, and interaction terms involving hydrolysis time had negligible effects. The regression coefficients indicated the effects of proteases on ACE-inhibitory activity were complex, binary interactions (A*T, A*F, T*F) positively impacted the formation of ACE-inhibitory peptide, whereas the ternary interaction A*T*F exerted an adverse effect due to the aminopeptidase and carboxypeptidase from Flavourzyme degrading the ACE-inhibitory peptide by cleaving terminal amino acids.

For the bitterness, individual protease, as well as A*T, A*F, and A*T*F were highly significant factors, A*H, A*F*H, and A*T*F*H were significant contributors. Negative regression coefficients indicated A*T*F and A*T*F*H reduced DSPH bitterness. Due to the cleavage specificity of protease, the hydrolysis reaction catalyzed by Alcalase or trypsin would increase with hydrolysis time until reaching a steady state, leading to the accumulation of bitter peptides at a maximum level. Flavourzyme, acting as an endo- and exoprotease, continuously released free amino acids from N- and C-terminal sites [37] and proceeded until proteins or peptides were extensively degraded. Therefore, prolonging the hydrolysis time was beneficial for debittering.

**Figure 1:**
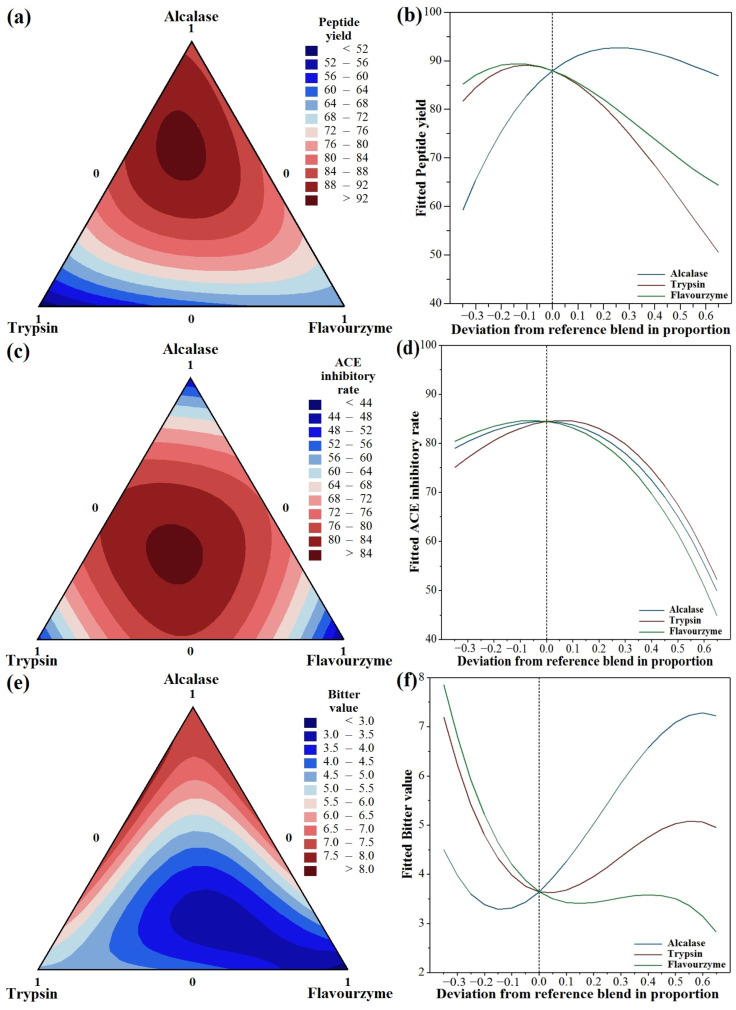
Mixture contour plots and response trace plot for preparation of low-bitter ACE-inhibitory peptides from sesame protein using triple-enzyme hydrolysis at 5 h. (**a**) Mixture contour plot of peptide yield, (**b**) response trace plot of peptide yield, (**c**) mixture contour plot of ACE-inhibitory rate, (**d**) response trace plot of ACE-inhibitory rate, (**e**) mixture contour plot of bitter value, (**f**) response trace plot of bitter value. The reference blend set at centroid of the mixture design, with equal proportions of Alcalase, trypsin, and Flavourzyme (0.33:0.33:0.33).

Ternary contour plots were constructed to illustrate the effects of Alcalase, trypsin, and Flavourzyme on the formation of low-bitter ACE-inhibitory peptides (Figure 1). It was seen from Figure 1a that high peptide yields appeared at the regions with a high Alcalase ratio. Figure 1c highlighted the high ACE-inhibitory activity was observed when Alcalase, trypsin and Flavourzyme were present in approximately equal proportions. The low bitter value was mainly associated with regions enriched in Flavourzyme (Figure 1e). The proportions of Alcalase, trypsin, and Flavourzyme for optimal responses (peptide yield, ACE-inhibitory activity, and bitter value) were divergent (Figure 1b,d,f).

To identify the best overall conditions, the composite desirability (D), defined as the geometric mean of individual response desirabilities [38], was used to identify the optimal protease ratio for simultaneous optimization. A D value closer to 1 indicates a better overall match to the target criteria. By maximizing the geometric mean of the individual desirabilities (d) for peptide yield, ACE-inhibitory activity, and bitter value, an optimized D of 0.99 was obtained. The corresponding protease composition was 33.20% Alcalase, 31.31% trypsin, and 35.48% Flavourzyme, with an optimal hydrolysis time of 4.82 h.

### 3.2. Optimization of Hydrolysis Conditions for Preparation of Low-Bitter ACE-Inhibitory Peptide Using RSM

The RSM models were validated based on low *p* values (*p* < 0.01), insignificant lack of fit (*p* > 0.05), and high R^2^ values (Table 4). These models were suitable for interpreting the actual relationship between hydrolysis factors (E/S, pH, and Temperature) and the preparation of low-bitter ACE-inhibitory peptide (peptide yield (Y_1_), ACE inhibitory rate (Y_2_), and bitter value (Y_3_)), and for optimizing the corresponding hydrolysis conditions using triple-enzyme system. Figure S2 visualized the effects of key hydrolysis factors on the peptide yield, ACE-inhibitory rate, and bitter value.

The uncoded regression equations of RMS models were expressed in the following equations: Y_1_ = −676.50 + 0.01 X_1_ + 155.70 X_2_ + 2.72 X_3_ − 9.3 × 10^−7^ X_1_^2^+ 2 × 10^−4^ X_1_X_2_ + 7.1 × 10^−5^ X_1_X_3_ − 9.43 X_2_^2^ + 0.06 X_2_X_3_ − 0.04 X_3_^2^, Y_2_ = −1004.41 + 0.03 X_1_ + 172.89 X_2_ + 10.87 X_3_ − 2.38 × 10^−6^X_1_^2^ + 3.62 × 10^−4^ X_1_X_2_ + 1.28 × 10^−4^ X_1_X_3_ − 10.30 X_2_^2^ − 0.03 X_2_X_3_ − 0.12 X_3_^2^, Y_3_ = 94.6644 − 0.0023 X_1_ − 14.09 X_2_ − 1.15 X_3_ + 1.65 × 10^−7^ X_1_^2^ − 3.6 × 10^−5^ X_1_X_2_ + 5.33 × 10^−6^ X_1_X_3_ + 0.81 X_2_^2^ + 0.02 X_2_X_3_ + 0.01 X_3_^2^.

As shown in Figure S2d, the optimal conditions for peptide yield, ACE-inhibitory activity, and bitter value did not coincide; therefore, the D calculated by Minitab 17 was used to identify the most balanced conditions for the three responses. The highest D of 0.9445 corresponded to an E/S ratio of 126,793.03 nkat/g, pH8.40, and 45 °C (Figure S2e). Under the optimized conditions, the predicted peptide yield, ACE-inhibitory rate, and bitter value were at 93.26%, 95.99%, and 3.20, respectively. The parallel tests yielded values of 93.19 ± 0.14%, 95.92 ± 0.23%, 3.15 ± 0.09, all within 95% confidence interval of the predictions (*p* values were 0.435, 0.626, 0.39, respectively), thereby confirming the reliability of the RSM models.

### 3.3. Fractionation of DSPH by Ultrafiltration

Table 5 illustrates that the DSPH-V showed higher ACE-inhibitory activity than other fractions, aligning with previous findings that shorter peptides generally displayed strong ACE-inhibitory activity [39,40]. Due to smaller size, these short peptides could more readily access and bind to the active pockets of ACE, thereby modulating its activity.

However, bitter value increased with decreasing molecular weight of the ultrafiltration fractions, peaking between 2 kDa and 3 kDa. The bitterness of DSPH-V was higher than that of DSPH and its other fragments. This result was supported by Iwaniak’s finding that soybean peptides with the molecular weight (MW) between 1.9 and 3.3 kDa exhibited higher bitterness than peptides outside this range [41]. Long peptides may have strong steric hindrance [42] and adopt folded conformations that reduce their interaction with bitter receptors, resulting in lower bitterness. Short peptides would either fail to adopt the specific conformation required to activate bitter receptors [43] or inhibit bitterness by blocking these receptors [44]. Considering the ACE-inhibitory activity and bitterness, DSPH-VI was selected for further purification to isolate low-bitter ACE-inhibitory peptides.

### 3.4. Purification of DSPH-VI by Medium-Pressure Chromatography System

**Figure 2:**
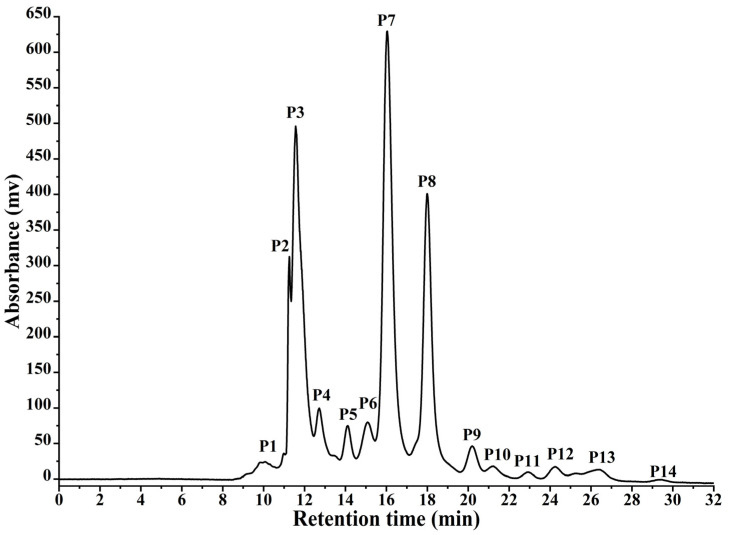
The elution profile of DSPH-VI separated by medium-pressure chromatography. P1–P14 represent peptide fractions corresponding to different chromatographic peaks, and these peaks were numbered sequentially with increasing retention time.

Figure 2 shows 14 peaks in the medium-pressure chromatography elution profile of DSPH-VI, including four distinct peaks (P2, P3, P7, P8). The characteristics of these chromatographic peaks are presented in Table 6. Fractions P11 and P14 exhibited the highest ACE-inhibitory activity, whereas fractions P1 and P2 had the lowest bitterness. This phenomenon was likely associated with the retention mechanism of RP-HPLC, in which compounds eluted from strong to weak polarity.

The hydrophobicity of peptide influences the ACE-inhibitory activity and bitterness; e.g., Wu and Aluko [45] highlighted the roles of hydrophobic, bulky, and acidic amino acids in enhancing the ACE-inhibitory activity. Hydrophobic residues (e.g., Val, Leu, Ile) could penetrate deeply into the S1, S1′, and S2 hydrophobic pockets of ACE through the hydrophobic interaction [46,47]. Bulky aromatic residues (e.g., Phe, Trp, Tyr) effectively occupied the active-site cavity and formed extensive hydrophobic and π–π interactions with aromatic residues (e.g., His, Tyr) in ACE, stabilizing the peptide–enzyme complex [48,49]. Acidic residues enabled the coordination of the catalytic Zn^2+^ ion through their negatively charged carboxyl groups, locking the metal center in a non-productive state [49,50,51]. These interactions enhanced the ACE-inhibitory activity of peptides.

Bitterness is likewise associated with peptide hydrophobicity and sequence. Bulky hydrophobic amino acids at the C-terminus and basic amino acids at the N-terminus correlated with bitterness [52]. Bulky hydrophobic residues at the C-terminus fitted into the hydrophobic pockets of T2Rs, which were enriched in nonpolar residues, thereby strengthening hydrophobic and van der Waals interactions and stabilizing the peptide–receptor complex [41,53,54]. In contrast, N-terminal basic residues enhanced bitterness mainly through electrostatic and hydrogen bond interactions with negatively charged or polar groups on the T2Rs surface [55]. Together, a basic, hydrophilic N-terminus and a hydrophobic C-terminus conferred pronounced amphiphilicity, facilitating peptide partitioning near the cell membrane and insertion into the receptor binding pocket in a favorable orientation [33,54]. Consequently, fractions with longer retention time tended to exhibit stronger ACE-inhibitory activity and more intense bitterness. Given the ACE-inhibitory activity, bitterness, and relative abundance, the peptides in fraction P7 were selected for identification using Nano LC–MS/MS.

### 3.5. Identification of Low-Bitter ACE-Inhibitory Peptide from the Fraction P7

Table 7 summarizes 17 peptides from fraction P7 identified by Nano LC-MS/MS, with molecular weights between 611.38–677.39 Da. The ACE-inhibitory peptides characterized in this study differed entirely from those reported by Wang et al. and Lu et al. [7,8]. The peptides identified in this paper were generally smaller than those produced by sequential hydrolysis of sesame protein with pepsin, trypsin, and **α**-chymotrypsin [7], or by dual-enzyme hydrolysis consisting of Alcalase and trypsin [8]. These discrepancies in peptide profiles are likely attributable to difference in enzymolysis conditions and separation techniques. The broad specificity of Flavourzyme facilitated the breakdown of sesame protein into shorter peptides, thereby favoring the generation of smaller ACE-inhibitory peptides. Moreover, these peptides identified in this work were originated from the permeate solution obtained using the 1000 Da MWCO ultrafiltration membrane. In contrast, previously identified peptides were obtained from the fraction separated by the 3000 Da MWCO ultrafiltration membrane [7,8]. Consequently, these factors resulted in a distinct set of ACE-inhibitory peptides from DSPH in the present study.

Compared with other identified peptides, APQLGR, LYVTR, YELPR, FPLAGR, and APWLR showed higher ACE-inhibitory activity, likely due to the presence of basic amino acids at the C-terminus [56]. Accordingly, the C-terminal R probably enhanced the ACE inhibitory activities of these peptides. Additionally, the contribution of Leu (L) to ACE-inhibitory activity should be emphasized, e.g., the ACE-inhibitory peptides with N-terminal L accounted for 13.30% of the entries in the BIOPEP database (https://biochemia.uwm.edu.pl/biopep-uwm/ (accessed on 10 November 2024)), and the C-terminal L has been shown to strengthen the ACE inhibitory activity [57]. Notably, L frequently appears in the internal positions of ACE-inhibitory peptides, including GLDIQK [58], VLKP [59], MDLA [60], indicating that L contributed to the ACE inhibitory activities of LYVTR, APGLGR, YELPR, FPLAGR, and APWLR. Moreover, Pro (P) was regarded as a feature of ACE-inhibitory peptides. Its rigid pyrrolidine ring was thought to help orient the peptide optimally within the ACE active site, thereby enhancing inhibitory [61].

Consistent with other naturally derived ACE-inhibitory peptides, the IC_50_ values of the newly identified peptides, APWRL (2.41 μM) and APQLGR (2.67 μM), were approximately three orders of magnitude higher than that of the synthetic antihypertensive drug lisinopril (typically in the nM range [62]). Nevertheless, their inhibitory potencies were notably stronger than those of some well-established food-derived peptides, such as VPP (IC_50_ = 9 μM) and IPP (IC_50_ = 5 μM) [63]. These findings suggested that although these peptides are unlikely to achieve pharmaceutical-grade efficacy, they are promising candidates for incorporation into functional foods or nutraceutical formulations to promote cardiovascular health.

Table 7 shows most of the identified peptides obeyed the Q rule, a tool used to preliminarily predict the bitterness of peptide based on average hydrophobicity [54]. According to the rule, bitter peptides typically exhibit Q values above 1400 cal/mol, while the non-bitter peptides exhibit Q values below 1300 cal/mol. Values between these thresholds are undetermined [41]. However, APLLAR and APWLR were exceptions.

The Q rule primarily correlates between peptide bitterness and the overall hydrophobicity of constituent amino acids, neglecting structural factors (steric effect, electrostatic distribution, the binding sites of bitter receptors, etc.) that also affect the taste performance [13,22,41]. For example, bulky hydrophobic amino acids at the C-terminus and basic amino acids at the N-terminus were highly correlated with bitterness [55]. Yu et al. found ADW reduced bitterness intensity by blocking T2R14 through simultaneous interactions with Thr86, Asp168, and Phe247 in the T2R14 [22]. Hence, exclusive reliance on the Q rule may lead to inaccurate predictions. Some identified peptides exhibited higher bitter values than fraction P7 as a whole, suggesting that specific components in fraction P7 may block the bitter receptor.

Given strong the ACE-inhibitory activity, low bitterness, and abundance of precursor proteins, APQLGR and APWLR were compelling candidates for investigation. Elucidating their mechanisms in inhibiting ACE and blocking bitter taste receptors was essential for advancing the understanding of structure–activity relationships and guiding the development of functional ingredients targeting cardiovascular health and sensory quality.

### 3.6. Molecular Docking for Peptides and ACE

**Figure 3:**
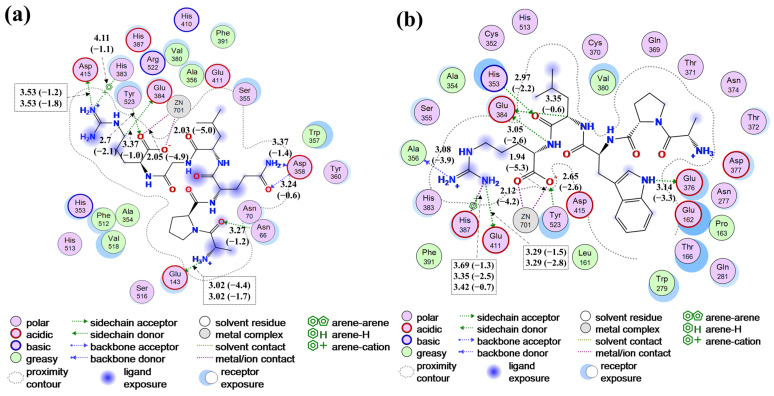
Molecular docking for APQLGR and APWLR with ACE (PDB: 1O86). (**a**) Receptor–ligand interaction between APQLGR and ACE, (**b**) receptor–ligand interaction between APWLR and ACE. Magenta, green, and red contours in (**a**,**b**) represent polar, hydrophobic, and exposed field, respectively. Data and date in the brackets are bond length and bond Energy (kcal/mol) in (**a**,**b**).

It has been reported that ACE contains three active pockets (S1, S2, and S1’): S1 contains Ala354, Glu384, and Tyr523 residues; S2 consists of Gln281, His353, Lys511, His513, and Tyr520 residues; and S1’ is composed of Glu162 residue. Moreover, ACE activity is critically governed by the tetrahedral coordination of the catalytic Zn^2+^ center (Zn^2+^ ion, His383, His387, and Glu411) [64]. The interaction between APQLGR and ACE is illustrated by Figure S3a and Figure 3a. The absence of prominent red contours at the protein–ligand interface indicated that APQLGR was deeply embedded in ACE. The Ala1, Gln3, and Arg6 of APQLGR bound to ACE by (i) hydrogen bond with Glu143, Asp358, Glu384, Asp415, Asn66, Tyr523, (ii) metal/ionic bond with Zn701, Glu143, Asp415, (iii) cation-π bond with His383. These interactions between APQLGR and ACE involved the S1 pocket (Glu384, Tyr523) and Zn^2+^ Zone (Zn701 and His383), thereby impacting ACE activity.

As illustrated in Figure S3b, APWLR was located near the Zn^2+^ ion and deeply buried in the ACE. This peptide formed interactions through Trp3, Leu4, and Arg5, which bound to the S1 pocket (Glu384, Tyr523), S2 pocket (His353), and the Zn^2+^ zone (Zn701, His387, Glu411). Additionally, APWLR affected the ACE by forming hydrogen bonds with Ala356 and Glu376 (Figure 3b). Compared with APQLGR (binding energy of −26.4 kcal/mol), APWLR demonstrated stronger binding affinity (−33.5 kcal/mol), providing a structural basis for its higher ACE-inhibitory activity.

### 3.7. Binding Interaction Between Peptides and T2R14

**Figure 4:**
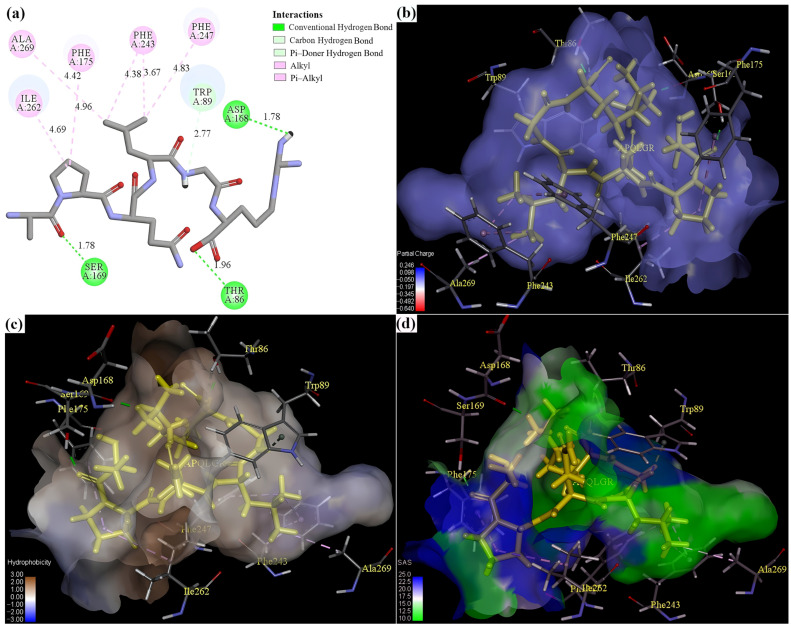

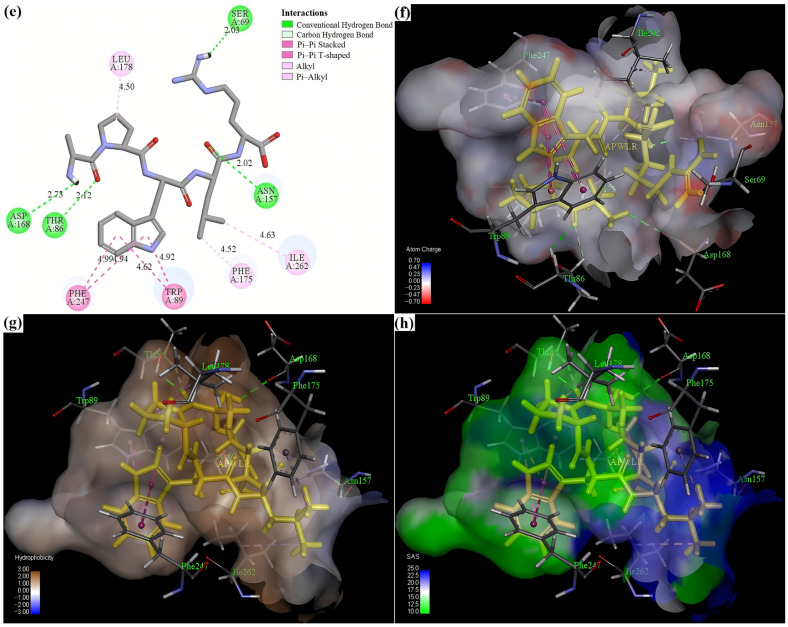
The docking interactions of APQLGR and APWLR with T2R14. (**a**) The 2D plot of the interaction between APQLGR and T2R14, (**b**) the atom charge surface at the binding site of APQLGR–T2R14, (**c**) the hydrophobicity surface at the binding site of APQLGR–T2R14, (**d**) the solvent accessibility Surface (SAS) at the binding site of APQLGR–T2R14, (**e**–**h**) the 2D interaction plot, and atom charge surface, hydrophobicity surface, SAS of APWLR–T2R14, respectively. The number in (**a**,**e**) is the bond length; the surface in (**b**,**f**) colored by atom charge of the receptor residue, with negative electricity in red and positive electricity in blue; color contours in (**c**,**g**) represent the hydrophobicity of the receptor residue, from blue for hydrophilic to brown for hydrophobic; color contours in (**d**,**h**) express the solvent accessibility of the receptor residues, from blue for exposed to green for buried.

The binding behaviors of APQLGR and APWLR with T2R14 are illustrated in Figure S4 and Figure 4. Both peptides exhibited strong affinity for T2R14, embedding deeply within the binding pocket and forming non-covalent interaction networks with surrounding amino acid residues.

Despite their comparable binding affinities, the two peptides displayed distinct interaction profiles with T2R14. The interaction between APQLGR and T2R14 involved (i) hydrogen bond to Ser169, Thr86, Asp168, and Trp89, (ii) Alkyl/π–Alkyl bond to Phe247, Phe243, Phe175, Ala269, and Ile262 (Figure 4a). In contrast, the APWLR–T2R14 complex featured hydrogen bonds between Ala1 and Leu4 of APWLR and Asp168, Thr86, Asn157, and Ser69 of T2R14, with bond distances of 2.73 Å, 2.12 Å, 2.02 Å, 2.03 Å, respectively. Additionally, the Trp3 of APWLR established π–π stacked/T–shaped interactions with Phe24 and Trp89 (4.99 Å and 4.94 Å, 4.62 Å and 4.92 Å, respectively). Furthermore, AWLR connected with Phe175, Ile262, and Leu178 via Alkyl/π–Alkyl interaction (4.52 Å, 4.63 Å, and 4.50 Å; Figure 4e).

In addition to the specific bonds identified, electrostatic effect, hydrophobic interaction, and other structural characteristics also contributed to the binding orientation and overall interaction patterns. Electrostatic potential mapping revealed that the electrostatic interactions between APQLGR and T2R14 (Figure 4b) were considerably stronger than those observed for the APWLR–T2R14 complex (Figure 4f). This difference arose from the polar Gln3 in APQLGR, which carries a higher partial charge than the nonpolar Trp3 in APWLR, allowing APQLGR to interact more favorably with the polar residues within the T2R14 binding pocket.

Conversely, the hydrophobic interaction analysis revealed an opposite trend (Figure 4c,g). APWLR was enveloped by highly hydrophobic contours (brown regions), whereas APQLGR resides in areas of lower hydrophobicity (white regions). Their hydrophobicity values (Q = 1900 cal/mol for APWLR vs. Q = 1066.67 cal/mol for APQLGR) supported this trend. The strong hydrophobicity of APWLR guided it towards the nonpolar residues within the T2R14 pocket. Consequently, the two peptides adopted distinct conformations and occupied different regions in the T2R14. The Pro2 and Arg6 of APQLGR remained exposed on the surface of the binding pocket (Figure 4d), whereas most of APWLR was buried deeply within T2R14, except for Arg5 (Figure 4h).

The distinct binding modes of APQLGR and APWLR implicated specific amino acid residues as critical for T2R14 modulation. T2R14 is a broadly tuned receptor, activated by various bitter substances through binding key residues including Thr86, Trp89, Leu178, Phe243, Phe247, Ile262 [65,66,67]. The bitter blockers engage these sites by directly competition with bitter agonists or by inducing allosteric inhibition upon receptor binding, thereby attenuating bitterness perception [68,69,70]. For instance, the T2R14 blocker QRPR docked with the Asn157, Asp168, Leu178, and Ile262 [71]; ADM and ADW inhibited T2R14 by binding with Thr86, Asp168, and Phe247 [22]. Integrating above docking results and these previous findings, it could be inferred that Thr86, Trp89, Asp168, Phe243, Phe247, and Ile262 would play the main role in blocking T2R14 by APQLGR, while Thr86, Trp89, Asn157, Asp168, Leu178, Phe247, and Ile262 are probably crucial for T2R14 blockade by APWLR.

**Figure 5:**
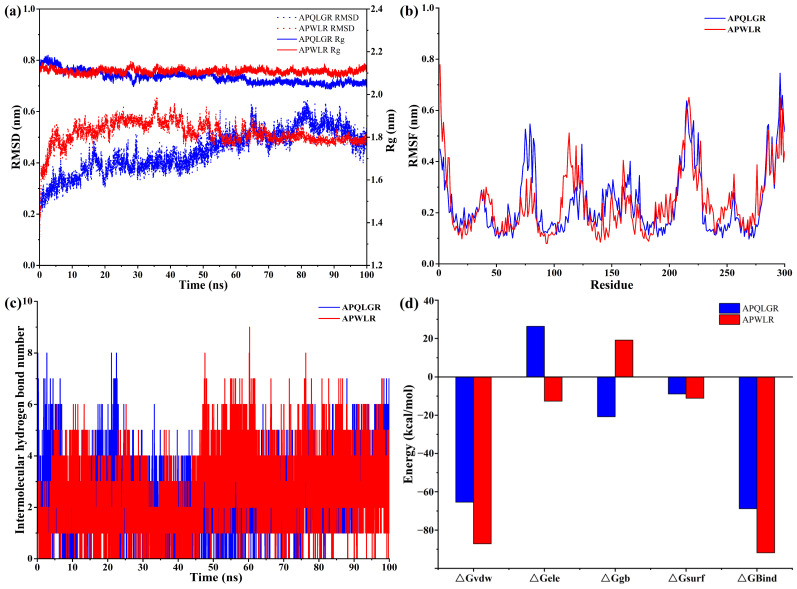
The molecular dynamics simulation of APQLGR–T2R14 and APWLR–T2R14. (**a**) The RMSD and Rg of both peptide–T2R14 complexes, (**b**) the RMSF of both peptide–T2R14 complexes, (**c**) hydrogen bonds binding between peptides and T2R14 during molecular dynamics period, (**d**) binding free energies of both peptide–T2R14 complexes. The ΔGvdw, ΔGele, ΔGgb, ΔGsurf, and ΔGbind in (**d**) represent the van der Waals energy, electrostatic energy, polar solvent energy, non-polar solvent energy, total binding free energy, respectively.

To validate the stability of the docked poses and further explore their interaction dynamics, 100 ns MD simulations were performed for both peptide–T2R14 complexes. The stability of the complexes along the simulation trajectory was evaluated by monitoring RMSD and Rg (Figure 5a). It is well-established that a smooth, converged RMSD curve indicates a stable ligand–receptor complex, and the smaller Rg value reflects the more compact and tightly folded structure [24,25]. For both the APQLGR–T2R14 and APWLR–T2R14 systems, their RMSD curves remained stable, fluctuating within a narrow range of less than 0.4 nm throughout the MD simulation. Consistently, the Rg values also remained steady within minor fluctuations ranging from 2.05 to 2.15 nm. These results indicated that both peptides form stable, compact complexes with T2R14, confirming the reliability of the initial docking poses.

To evaluate the receptor’s dynamic behavior upon peptide binding, the flexibility of individual amino acid residues was quantified by RMSF [24,25]. As illustrated in Figure 5b, the RMSF curves of T2R14 differed markedly between the two complexes, indicating that each peptide uniquely modulates receptor flexibility. These distinct fluctuation patterns suggested that the formation pathways and the resulting dynamic conformational states of the APQLGR–T2R14 and APWLR–T2R14 complexes were different. This finding was consistent with the molecular docking results (Figure 4a,e), indicating the two peptides interacted with different sets of amino acid residues within the T2R14 binding pocket.

The distinct binding mechanisms of the two peptides were further elucidated by analyzing the hydrogen bond dynamics and the decomposition of binding free energies (Figure 5c,d). The energy components revealed pronounced differences between the two systems; for example, electrostatic interaction opposed binding in the APQLGR–T2R14 system, whereas it facilitated binding in the APWLR–T2R14 system. Conversely, polar salvation enhanced the binding between APQLGR and T2R14 but weakened the binding between APWLR and T2R14. These thermodynamic divergences could be attributed to the key sequence difference between APQLGR and APWLR: the hydrophilic, polar Gln3 residue in APQLGR versus the hydrophobic, non-polar Trp3 in APWLR. This difference altered how each peptide interacted with the electrostatic and hydrophobic microenvironment of the binding pocket of T2R14, as supported by analyses of the electrostatic potential and hydrophobicity surfaces at the binding interface (Figure 4b,c,f,g).

Finally, the total binding free energy for APWLR was higher than that for APQLGR, indicating a stronger binding affinity for T2R14. This quantitative finding corroborated the qualitative observations from the initial docking and the structural stability analyses from MD simulations.

## 4. Conclusions

This study established a D–guided optimization strategy for producing low-bitter ACE-inhibitory peptides from sesame protein using a triple-enzyme hydrolysis system. The optimized enzymatic conditions yielded hydrolysates with a high peptide yield, strong ACE-inhibitory activity, and acceptably low bitterness.

From the optimized hydrolysate, 17 peptides were identified, among which APQLGR and APWLR exhibited the strongest ACE inhibition and the lowest bitterness. Molecular docking indicated that both peptides effectively interact with the ACE active sites and the Zn^2+^ coordination region, albeit through distinct binding modes. Docking and MD simulations with T2R14 suggested that APQLGR and APWLR act as antagonists of T2R14, with peptide-specific stabilization mechanisms influenced by different energetic contributions.

In conclusion, these findings provide a practical strategy for transforming sesame protein into highly active, low-bitter functional ingredients, and this approach could also apply to the high-value utilization of other edible proteins. As APQLGR and APWLR appear to be promising candidates for development as ACE-inhibitory functional food components, future work should verify their stability and efficacy in vivo, refine structure–activity relationships, and interact with other T2Rs to elucidate the mechanisms underlying their favorable sensory profiles.

## Figures and Tables

**Table 1:** Matrix of the simplex-lattice mixture design for optimizing the ratio of triple-enzyme to produce low-bitter ACE-inhibitory peptide.

Run	Alcalase (A)/nkat/g	Trypsin (T)/nkat/g	Flavourzyme (F)/nkat/g	Hydrolysis Time (H)/h	Peptide Yield (Y_1_)/%	ACE Inhibitory Rate (Y_2_)/%	Bitter Value (Y_3_)
1	103,354 (1.00) ^1^	0 (0)	0 (0)	2.50 (−1)	83.08 ± 0.15	46.81 ± 0.18	5.98 ± 0.08
2	0 (0)	103,354 (1.00)	0 (0)	2.50 (−1)	46.67 ± 0.22	48.19 ± 0.37	4.31 ± 0.11
3	0 (0)	0 (0)	103,354 (1.00)	2.50 (−1)	58.97 ± 0.17	41.29 ± 0.29	2.56 ± 0.14
4	51,677 (0.50)	51,677 (0.50)	0 (0)	2.50 (−1)	83.76 ± 0.13	76.26 ± 0.41	6.16 ± 0.10
5	51,677 (0.50)	0 (0)	51,677 (0.50)	2.50 (−1)	81.45 ± 0.18	73.18 ± 0.53	5.08 ± 0.17
6	0 (0)	51,677 (0.50)	51,677 (0.50)	2.50 (−1)	59.56 ± 0.09	75.61 ± 0.48	3.78 ± 0.07
7	34,451.33 (0.33)	34,451.33 (0.33)	34,451.33 (0.33)	2.50 (−1)	87.34 ± 0.14	79.94 ± 0.35	3.52 ± 0.08
8	69,247.18 (0.67)	175,070.18 (0.17)	175,070.18 (0.17)	2.50 (−1)	89.92 ± 0.25	73.65 ± 0.29	4.69 ± 0.15
9	175,070.18 (0.17)	69,247.18 (0.67)	175,070.18 (0.17)	2.50 (−1)	72.98 ± 0.19	76.03 ± 0.43	3.85 ± 0.12
10	175,070.18 (0.17)	175,070.18 (0.17)	69,247.18 (0.67)	2.50 (−1)	75.29 ± 0.26	71.97 ± 0.34	3.09 ± 0.09
11	103,354 (1.00)	0 (0)	0 (0)	5.00 (1)	87.04 ± 0.11	48.04 ± 0.14	7.23 ± 0.13
12	0 (0)	103,354 (1.00)	0 (0)	5.00 (1)	48.99 ± 0.18	49.66 ± 0.35	4.95 ± 0.06
13	0 (0)	0 (0)	103,354 (1.00)	5.00 (1)	63.75 ± 0.14	42.51 ± 0.26	2.69 ± 0.16
14	51,677 (0.50)	51,677 (0.50)	0 (0)	5.00 (1)	86.08 ± 0.27	80.47 ± 0.48	7.59 ± 0.08
15	51,677 (0.50)	0 (0)	51,677 (0.50)	5.00 (1)	83.07 ± 0.23	75.72 ± 0.24	6.89 ± 0.09
16	0 (0)	51,677 (0.50)	51,677 (0.50)	5.00 (1)	60.92 ± 0.15	78.82 ± 0.31	4.34 ± 0.12
17	34,451.33 (0.33)	34,451.33 (0.33)	34,451.33 (0.33)	5.00 (1)	87.92 ± 0.12	82.74 ± 0.42	3.91 ± 0.07
18	69,247.18 (0.67)	175,070.18 (0.17)	175,070.18 (0.17)	5.00 (1)	91.06 ± 0.14	76.62 ± 0.38	5.86 ± 0.10
19	175,070.18 (0.17)	69,247.18 (0.67)	175,070.18 (0.17)	5.00 (1)	73.98 ± 0.16	80.53 ± 0.51	4.26 ± 0.13
20	175,070.18 (0.17)	175,070.18 (0.17)	69,247.18 (0.67)	5.00 (1)	77.34 ± 0.23	75.38 ± 0.27	3.52 ± 0.06

^1^ The number in brackets represents the coded value.

**Table 2:** RSM design and responses of dependent indexes to the hydrolysis factors.

Run	E/S (X_1_) /nkat/g	pH (X_2_)	Temperature (X_3_) /°C	Peptide Yield (Y_1_)/%	ACE Inhibitory Rate (Y_2_)/%	Bitter Value (Y_3_)
1	100,020 (−1) ^1^	7.7 (−1)	45 (0)	84.06 ± 0.36	81.91 ± 0.22	3.62 ± 0.03
2	150,030 (1)	7.7 (−1)	45 (0)	86.97 ± 0.13	87.65 ± 0.37	4.03 ± 0.05
3	100,020 (−1)	9.0 (1)	45 (0)	86.59 ± 0.25	83.69 ± 0.45	3.82 ± 0.04
4	150,030 (1)	9.0 (1)	45 (0)	90.28 ± 0.39	90.84 ± 0.51	4.09 ± 0.09
5	100,020 (−1)	8.35 (0)	40 (−1)	87.15 ± 0.30	83.22 ± 0.42	3.52 ± 0.06
6	150,030 (1)	8.35 (0)	40 (−1)	90.08 ± 0.24	86.46 ± 0.34	3.86 ± 0.10
7	100,020 (−1)	8.35 (0)	50 (1)	88.82 ± 0.19	86.17 ± 0.21	3.69 ± 0.12
8	150,030 (1)	8.35 (0)	50 (1)	93.89 ± 0.33	93.24 ± 0.30	4.19 ± 0.09
9	125,025 (0)	7.7 (−1)	40 (−1)	85.72 ± 0.28	84.99 ± 0.22	3.57 ± 0.05
10	125,025 (0)	9.0 (1)	40 (−1)	88.53 ± 0.44	88.41 ± 0.38	3.65 ± 0.08
11	125,025 (0)	7.7 (−1)	50 (1)	87.29 ± 0.31	88.35 ± 0.29	3.79 ± 0.14
12	125,025 (0)	9.0 (1)	50 (1)	90.84 ± 0.27	91.36 ± 0.36	4.14 ± 0.07
13	125,025 (0)	8.35 (0)	45 (0)	93.25 ± 0.15	95.81 ± 0.17	3.16 ± 0.12
14	125,025 (0)	8.35 (0)	45 (0)	93.01 ± 0.18	95.98 ± 0.39	3.28 ± 0.09
15	125,025 (0)	8.35 (0)	45 (0)	92.89 ± 0.21	95.40 ± 0.35	3.09 ± 0.11

^1^ The number in brackets represents the coded value.

**Table 3:** Analysis of variance for mixture design of triple-enzyme system producing low-bitter ACE- inhibitory peptide.

Response Variable	Source	DF	Adi SS ^1^	Adi MS ^2^	F	*p*
Peptide Yield (Y_1_)	Model	13	3619.27	278.41	243.53	<0.01
	Linear	2	1499.68	749.84	655.90	<0.01
	AT	1	459.60	459.60	402.02	<0.01
	AF	1	107.26	107.26	93.82	<0.01
	TF	1	46.69	46.69	40.84	<0.01
	ATF	1	96.82	96.82	84.69	<0.01
	AH	1	7.81	7.81	6.83	0.04
	TH	1	2.87	2.87	2.51	0.16
	FH	1	12.85	12.85	11.24	0.02
	ATH	1	0.27	0.27	0.24	0.65
	AFH	1	2.57	2.57	2.25	0.18
	TFH	1	1.54	1.54	1.34	0.29
	ATFH	1	0.13	0.13	0.11	0.75
	Residual error	6	6.86			
	Total	19	3626.13			
	Model	13	3619.27	278.41	243.53	<0.01
	R^2^ = 0.99, R_adj_^2^ = 0.99					
	Y_1_ = 84.65 A + 48.17 T + 61.49 F + 73.77 AT + 35.64 AF + 23.51 TF + 223.20 ATF + 1.91 AH + 1.16 TH + 2.45 FH − 1.79 ATH − 5.52 AFH − 4.27 TFH − 8.15 ATFH					
ACE inhibitory rate (Y_2_)	Model	13	4166.57	320.51	141.94	<0.01
	Linear	2	62.51	31.25	13.84	0.01
	AT	1	1245.34	1245.34	551.53	<0.01
	AF	1	1201.97	1201.97	532.33	<0.01
	TF	1	1390.18	1390.18	615.68	<0.01
	ATF	1	28.47	28.47	12.61	0.01
	AH	1	0.68	0.68	0.30	0.60
	TH	1	1.55	1.55	0.69	0.44
	FH	1	0.95	0.95	0.42	0.54
	ATH	1	2.89	2.89	1.28	0.30
	AFH	1	0.59	0.59	0.26	0.63
	TFH	1	1.40	1.40	0.62	0.46
	ATFH	1	0.15	0.15	0.07	0.80
	Residual error	6	13.55	2.26		
	Total	19	4180.12			
	Model	13	4166.57	320.51	141.94	<0.01
	R^2^ = 0.99, R_ad_^j2^ = 0.98					
	Y_2_ = 47.4 A + 49.3 T + 42.0 F + 121.4 AT + 119.3 AF + 128.3 TF − 121 ATF + 0.6 AH + 0.9 TH + 0.7 FH + 5.8 ATH + 2.6 AFT + 4.1 TFH − 8.9 ATFH					
Bitter value (Y_3_)	Model	13	41.17	3.17	52.64	<0.01
	Linear	2	16.73	8.36	139.01	<0.01
	AT	1	1.97	1.97	32.79	<0.01
	AF	1	2.46	2.46	40.94	<0.01
	TF	1	0.24	0.24	4.03	0.09
	ATF	1	8.43	8.43	140.07	<0.01
	AH	1	0.86	0.86	14.22	0.01
	TH	1	0.20	0.20	3.38	0.12
	FH	1	0.01	0.01	0.14	0.73
	ATH	1	0.08	0.08	1.30	0.30
	AFH	1	0.43	0.43	7.09	0.04
	TFH	1	0.01	0.01	0.14	0.72
	ATFH	1	0.44	0.44	7.25	0.04
	Residual error	6	0.36	0.06		
	Total	19	41.53			
	Model	13	41.17	3.17	52.64	<0.01
	R^2^ = 0.99, R_adj_^2^ = 0.97					
	Y_3_ = 6.54 A + 4.59 T + 2.65 F + 4.83 AT + 5.40 AF + 1.69 TF − 65.85 ATF + 0.63 AH + 0.31 TH + 0.06 FH + 0.96 ATH + 2.25 AFH + 0.32 TFH − 14.98 ATFH					

^1^ Adj SS represents adjusted sums of squares. ^2^ Adj MS represents adjusted mean squares.

**Table 4:** Analysis of Variance for RSM models of peptide yield, ACE-inhibitory activity, and bitter value.

Source	DF	Peptide Yield (Y_1_)				ACE Inhibitory Rate (Y_2_)				Bitter Value (Y_3_)			
		SS ^1^	MS ^2^	F	*p*	SS	MS	F	*p*	SS	MS	F	*p*
X_1_	1	26.65	26.65	200.04	<1 × 10^−4^	67.28	67.28	122.85	1 × 10^−4^	0.29	0.29	46.90	1 × 10^−3^
X_2_	1	18.61	18.61	139.68	<1 × 10^−4^	16.25	16.25	29.66	2.8 × 10^−3^	0.06	0.06	9.66	0.03
X_3_	1	10.95	10.95	82.22	3 × 10^−4^	32.16	32.16	58.72	6 × 10^−4^	0.18	0.18	29.72	2.8 × 10^−3^
X_1_^2^	1	16.17	16.17	121.37	1 × 10^−4^	105.93	105.93	193.42	<1 × 10^−4^	0.51	0.51	82.27	3 × 10^−4^
X_1_X_2_	1	0.15	0.15	1.14	0.33	0.50	0.50	0.91	0.38	0.00	0.00	0.80	0.41
X_1_X_3_	1	1.14	1.14	8.60	0.03	3.67	3.67	6.70	0.05	0.01	0.01	1.04	0.35
X_2_^2^	1	58.56	58.56	439.65	<1 × 10^−4^	69.91	69.91	127.65	<1 × 10^−4^	0.43	0.43	70.50	4 × 10^−4^
X_2_X_3_	1	0.14	0.14	1.03	0.36	0.04	0.04	0.08	0.79	0.02	0.02	2.96	0.15
X_3_^2^	1	3.49	3.49	26.22	3.7 × 10^−3^	35.51	35.51	64.84	5 × 10^−4^	0.27	0.27	43.04	1.2 × 10^−3^
Model	9	129.13	14.35	107.72	<1 × 10^−4^	305.10	33.90	61.90	1 × 10^−4^	1.61	0.18	29.06	9 × 10^−4^
Linear	3	56.20	18.73	140.64	<1 × 10^−4^	115.69	38.56	70.41	2 × 10^−4^	0.53	0.18	28.76	1.4 × 10^−3^
Quadratic	3	71.49	23.83	178.91	<1 × 10^−4^	185.21	61.74	112.73	<1 × 10^−4^	1.05	0.35	56.81	3 × 10^−4^
Cross Product	3	1.43	0.48	3.59	0.10	4.21	1.40	2.56	0.17	0.03	0.01	1.60	0.30
Error	5	0.67	0.13			2.74	0.55			0.03	0.01		
Lack of fit	3	0.60	0.20	5.94	0.15	2.56	0.85	9.60	0.10	0.01	0.00	0.44	0.75
Pure error	2	0.07	0.03			0.18	0.09			0.02	0.01		
Total	14	129.80				307.84				1.64			
R^2^		0.9949				0.9911				0.9812			
R_adj_^2^		0.9856				0.9751				0.9475			

^1^ SS represents sum of square. ^2^ MS represents mean square.

**Table 5:** The ACE inhibitory activity and bitter value of ultrafiltration fractions.

Fraction	DSPH	DSPH-I (>20 kDa)	DSPH-II (10–20 kDa)	DSPH-III (5–10 kDa)	DSPH-IV (3–5 kDa)	DSPH-V (2–3 kDa)	DSPH-VI (<1 kDa)
IC_50_ of ACE ^1^ (μg/mL)	28.75 ± 0.39 ^d^	78.34 ± 0.62 ^a^	49.93 ± 0.30 ^b^	34.89 ± 0.54 ^c^	26.13 ± 0.35 ^e^	20.41 ± 0.16 ^f^	15.68 ± 0.27 ^g^
Bitter value	3.15 ± 0.09 ^b^	1.62 ± 0.04 ^f^	1.97 ± 0.08 ^e^	2.45 ± 0.10 ^d^	3.08 ± 0.07 ^b^	3.41 ± 0.04 ^a^	2.74 ± 0.06 ^c^

^1^ The data with the same lowercase letter in the same row are not significantly different.

**Table 6:** The properties of fractions of DSPH-VI separated by medium-pressure chromatography system.

Fraction	Retention Time (min)	Proportion of Peak Area (%)	IC_50_ of ACE ^1^ (µg/mL)	Bitter Value
P1	10.02 ± 0.02	0.65 ± 0.03	20.45 ± 0.23 ^b^	0.94 ± 0.05 ^j^
P2	11.26 ± 0.01	4.48 ± 0.17	22.94 ± 0.17 ^a^	1.05 ± 0.08 ^j^
P3	11.56 ± 0.01	24.20 ± 0.29	18.03 ± 0.32 ^c^	1.69 ± 0.11 ^i^
P4	12.72 ± 0.01	4.70 ± 0.21	15.92 ± 0.45 ^d^	1.64 ± 0.07 ^i^
P5	14.12 ± 0.01	2.66 ± 0.08	13.55 ± 0.26 ^e^	1.97 ± 0.05 ^h^
P6	15.08 ± 0.01	3.96 ± 0.21	11.08 ± 0.11 ^f^	2.05 ± 0.12 ^h^
P7	16.05 ± 0.01	32.71 ± 0.38	10.31 ± 0.24 ^g^	1.99 ± 0.04 ^h^
P8	18.02 ± 0.01	20.38 ± 0.24	10.89 ± 0.21 ^f^	3.28 ± 0.11 ^g^
P9	20.22 ± 0.01	2.37 ± 0.11	11.05 ± 0.38 ^f^	3.97 ± 0.08 ^f^
P10	21.25 ± 0.01	0.98 ± 0.04	10.62 ± 0.09 ^fg^	4.69 ± 0.06 ^d^
P11	22.98 ± 0.01	0.48 ± 0.03	9.82 ± 0.14 ^h^	4.21 ± 0.09 ^e^
P12	24.28 ± 0.01	1.14 ± 0.03	13.27 ± 0.46 ^e^	5.84 ± 0.15 ^c^
P13	26.38 ± 0.02	1.08 ± 0.07	11.09 ± 0.29 ^f^	8.68 ± 0.13 ^b^
P14	29.48 ± 0.01	0.22 ± 0.02	10.16 ± 0.18 ^gh^	10.83 ± 0.09 ^a^

^1^ There is no significant difference between the data with the same lowercase letter in the same column.

**Table 7:** The physicochemical properties of peptides identified from the peak P7.

Peptide	RT (min)	Score	Mass (Da)	IC_50_ of ACE (μM)	Bitter Value	Q Value ^1^ (cal/mol)	Fragment of Proteins ^2^
APQLGR	14.44	99	640.37	2.67 ± 0.12	0.08 ± 0.02	1066.67	**Q2XSW6 (200–205)**, **A0A8M8USH5 (200–205)**
VPQLR	17.63	96	611.38	17.85 ± 0.17	1.31 ± 0.09	1472	A0A6I9SMJ1 (214–218), A0A6I9SWQ5 (182–186), A0A6I9TIB2 (40–44), A0A6I9TLE8 (18–22), A0A6I9TY30 (31–35), A0A6I9UGI4 (29–33), A0A6I9UNJ3 (182–186), A0A8M8V0Y9 (1879–1883), A0A8M8V376 (40–44), A0A8M8V679 (277–281)
LYVTR	18.5	99	650.38	2.98 ± 0.15	2.69 ± 0.07	1630	**Q2XSW7 (379–383)**, A0A6I9ULZ6 (65–69), A0A6I9UP68 (209–213), A0A8M8UUK6 (96–100)
EEFPR	19.12	99	676.32	11.57 ± 0.35	4.12 ± 0.05	1420	**Q8VX62 (72–76)**, A0A6I9TPH0 (67–71)
PNFRP	20.18	98	629.33	32.65 ± 0.26	1.23 ± 0.08	1722	A0A6I9TU65 (117–121), A0A6I9U9C4 (160–165), A0A8M8UUY4 (37–41), A0A8M8V327 (37–41), A0A8M8VAA7 (36–40), A0A6I9SU53 (112–116), A0A6I9TTE3 (124–128)
APLLAR	20.19	94	639.41	4.89 ± 0.23	0.23 ± 0.03	1608.33	A0A6I9SR92 (180–185), A0A6I9SM86 (219–224)
PQLLR	20.51	94	625.39	5.08 ± 0.21	1.38 ± 0.07	1618	A0A6I9TEW4 (111–115), A0A6I9TQG0 (471–475), A0A6I9TST8 (472–476), A0A6I9SV82 (20–24), A0A6I9T4H2 (43–47), A0A6I9T6U6 (556–560), A0A6I9T7S4 (146–150), A0A6I9TDK6 (43–47), A0A6I9TDM8 (53–58), A0A6I9U900 (328–332), A0A6I9UAT0 (5–9), A0A6I9URC1 (569–573), A0A8M8V0S7 (1004–1008), A0A8M8VBZ1 (1942–1946), A0A6I9SKX1 (111–115)
YELPR	20.92	98	676.35	2.33 ± 0.22	5.98 ± 0.03	1838	A0A6I9TBX1 (44–48), A0A6I9SV48 (55–59)
LMLRP	21.34	95	644.37	28.11 ± 0.38	1.05 ± 0.05	1898	A0A6I9U160 (81–85), A0A6I9TZF4 (561–565), A0A6I9U0A4 (565–569), A0A6I9SUE9 (3–7), A0A6I9T0I9 (247–251), A0A6I9U160 (81–85)
PELLR	21.56	99	626.38	23.43 ± 0.22	2.27 ± 0.04	1748	A0A6I9TDM0 (711–715), A0A6I9TXG1 (681–685), A0A8M8UR70 (711–715), A0A8M8UZK4 (515–519), A0A6I9SRS3 (574–578), A0A6I9STC9 (56–60), A0A6I9T7R1 (208–212), A0A6I9TAC5 (56–60), A0A6I9THK3 (187–191), A0A6I9TSU9 (738–742), A0A6I9TVX4 (725–729), A0A6I9UDG9 (165–169), A0A6I9UIJ3 (58–62), A0A6I9UL63 (394–398), A0A6I9TD67 (49–53)
DLKFR	21.71	99	677.39	14.45 ± 0.21	4.59 ± 0.07	1568	**Q2XSW7 (197–201)**, A0A6I9TFR6 (27–31), A0A6I9T718 (162–166), A0A6I9T8G1 (240–244), A0A6I9UM40 (71–75)
SHVLY	22.75	90	617.32	15.12 ± 0.12	0.91 ± 0.03	1504	A0A6I9TL88 (71–75), A0A6I9TIT7 (70–74), A0A8M8V406 (57–61), A0A6I9U5W2 (540–544), A0A6I9UA71 (539–543)
ADLFR	25.05	99	620.33	15.86 ± 0.19	2.53 ± 0.02	1414	A0A6I9T8B9 (494–498), A0A6I9TIS4 (861–865), A0A6I9SRU6 (469–473), A0A6I9TID6 (32–36), A0A6I9U2L3 (722–726), A0A6I9U9U1 (722–726), A0A6I9U2U5 (142–146), A0A6I9UA36 (98–102)
LDKPF	26.08	95	618.34	21.08 ± 0.19	2.87 ± 0.05	1946	A0A6I9T8Y4 (261–265), A0A6I9TKZ6 (348–352), A0A6I9UFJ1 (149–153)
FPLAGR	26.36	98	659.38	2.12 ± 0.11	3.22 ± 0.07	1525	**Q9AUD0 (531–536)**
APWLR	28.18	94	641.36	2.41 ± 0.07	0.05 ± 0.03	1900	A0A6I9ST89 (22–26), A0A6I9TCS7 (286–290)
AVPLLK	32.81	93	639.42	12.67 ± 0.27	6.25 ± 0.06	1897	A0A6I9T8P4 (435–440), A0A6I9T9X8 (488–493), A0A6I9T9Y4 (488–493), A0A6I9TC24 (477–482), A0A6I9U6B6 (615–620), A0A8M8VBH2 (609–614), A0A8M8VCG5 (615–620), A0A6I9UNY4 (579–584)

The IC_50_ value of lisinopril measured under the same conditions as those used for peptides was (4.92 ± 0.29) nM. ^1^ Q is defined as the average hydrophobicity of a peptide. The Q value is expressed in cal·mol^−1^ per residue (often written simply as cal/mol) and is calculated as: Q value = ∑Δg/n, where Δg is the hydrophobic free energy of each amino acid residue and n is the number of amino acid residues in the peptide. ^2^ Precursor proteins of identified peptides are retrieved from the UniProt database (Search term inputs *Sesamum indicum*), codes, the numbers in the brackets represent the entries in the UniProt database and the positions of peptides in their proteins, respectively. The bold codes signify the storage protein, like 2S, 7S, 11S sesame proteins, etc.

## Data Availability

The original contributions presented in the study are included in the article/Supplementary Materials. Further inquiries can be directed to the corresponding author.

## Supplementary Materials

The following supporting information can be downloaded at: https://www.mdpi.com/article/10.3390/foods15020279/s1, Figure S1: The representative picture of sesame (*Sesamum indicum* L.) seeds; Figure S2: RSM contour plots and desirability function for low-bitter ACE-inhibitory sesame peptide; Figure S3: Molecular docking for APQLGR and APWLR with ACE (PDB: 1O86); Figure S4: The 3D docking interaction of selected peptides with T2R14.
