# Supplementary material for: Preparation, Separation, and Identification of Low-Bitter ACE-Inhibitory Peptides from Sesame (Sesamum indicum L.) Protein
Source: Foods. 2026 Jan 12;15(2):279. doi: 10.3390/foods15020279 (PMC12841349; doi:10.3390/foods15020279)
Supplement: Supplementary file 1 [file foods-15-00279-s001.zip › Figure S2.pdf]

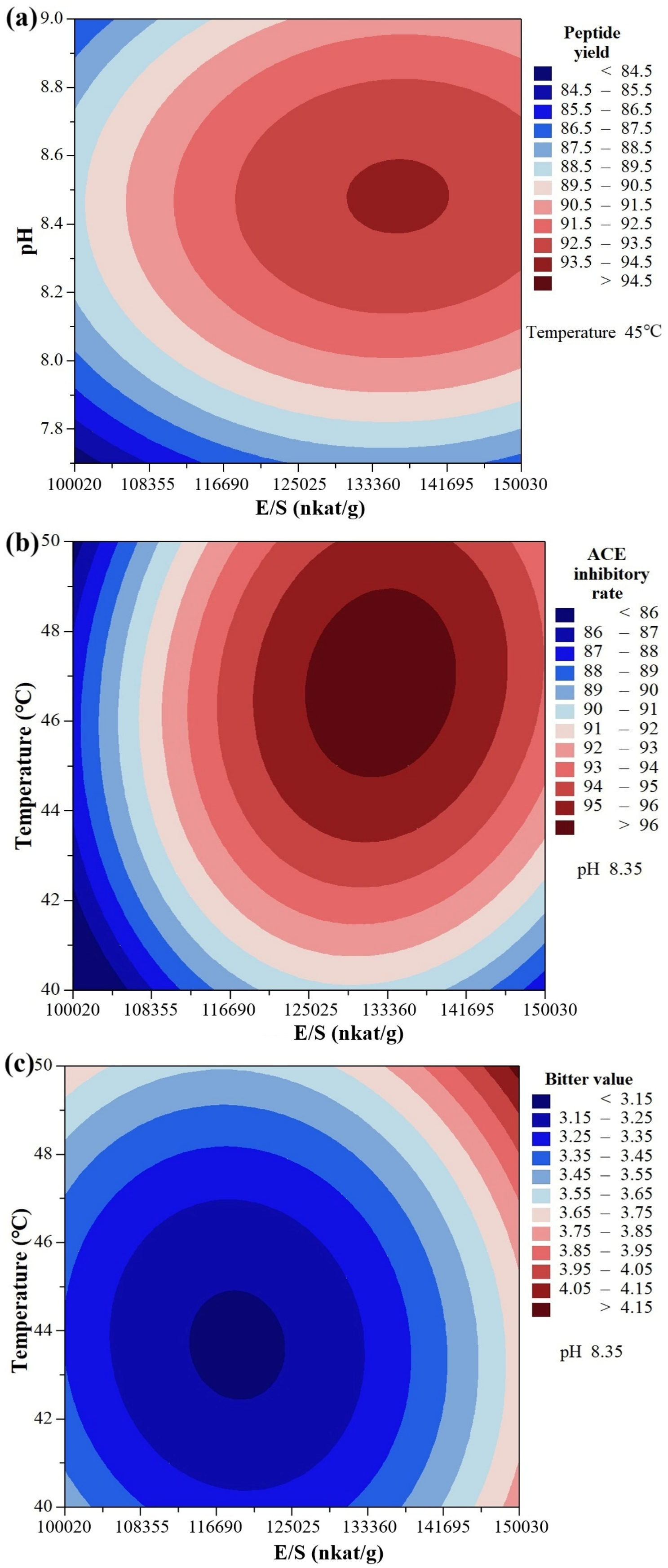

(a)
Peptide yield
< 84.5
84.5 – 85.5
85.5 – 86.5
86.5 – 87.5
87.5 – 88.5
88.5 – 89.5
89.5 – 90.5
90.5 – 91.5
91.5 – 92.5
92.5 – 93.5
93.5 – 94.5
> 94.5
Temperature 45°C
pH
9.0
8.8
8.6
8.4
8.2
8.0
7.8
100020 108355 116690 125025 133360 141695 150030
E/S (nkat/g)
(b)
ACE inhibitory rate
< 86
86 – 87
87 – 88
88 – 89
89 – 90
90 – 91
91 – 92
92 – 93
93 – 94
94 – 95
95 – 96
> 96
pH 8.35
Temperature (°C)
50
48
46
44
42
40
100020 108355 116690 125025 133360 141695 150030
E/S (nkat/g)
(c)
Bitter value
< 3.15
3.15 – 3.25
3.25 – 3.35
3.35 – 3.45
3.45 – 3.55
3.55 – 3.65
3.65 – 3.75
3.75 – 3.85
3.85 – 3.95
3.95 – 4.05
4.05 – 4.15
> 4.15
pH 8.35
Temperature (°C)
50
48
46
44
42
40
100020 108355 116690 125025 133360 141695 150030
E/S (nkat/g)

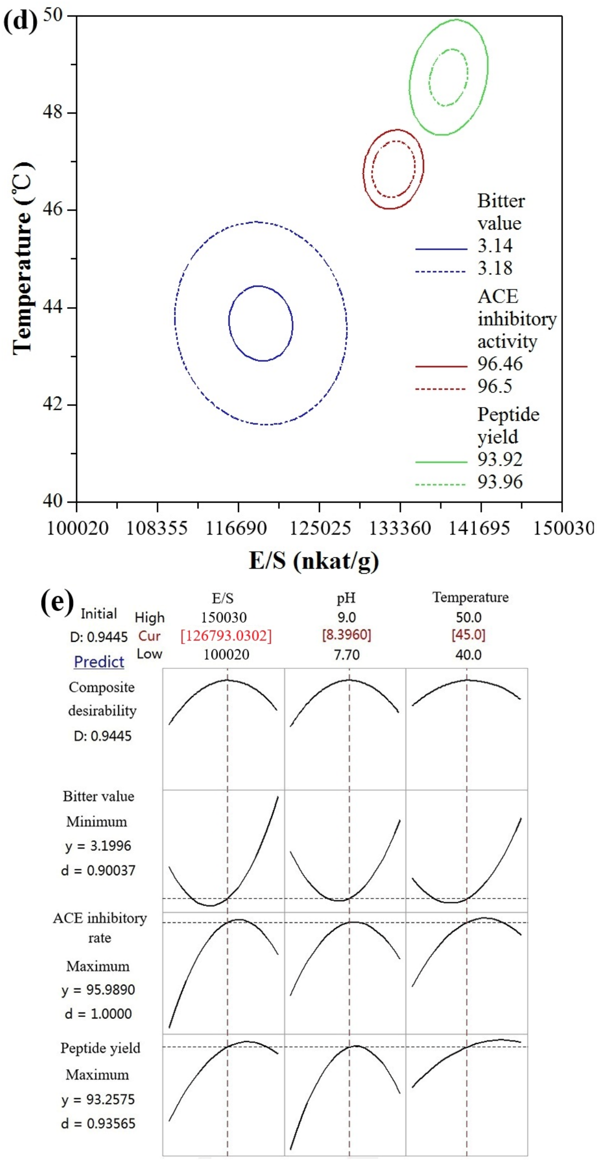

Figure S2. RSM contour plots and desirability function for low-bitter ACE-inhibitory sesame peptide. (**a**) Contour plot for the peptide yield build by the first and second significant factors E/S and pH, (**b**) Contour plot for ACE inhibitory rate constructed by the first and second significant factors E/S and Temperature, (**c**) Contour plot for bitter value by the first and second significant factors E/S and temperature, (**d**) overlaid contour plot for various response, (**e**) Composite desirability for these responses.
