# Supplementary material for: Preparation, Separation, and Identification of Low-Bitter ACE-Inhibitory Peptides from Sesame (Sesamum indicum L.) Protein
Source: Foods. 2026 Jan 12;15(2):279. doi: 10.3390/foods15020279 (PMC12841349; doi:10.3390/foods15020279)
Supplement: Supplementary file 1 [file foods-15-00279-s001.zip › Figure S3.pdf]

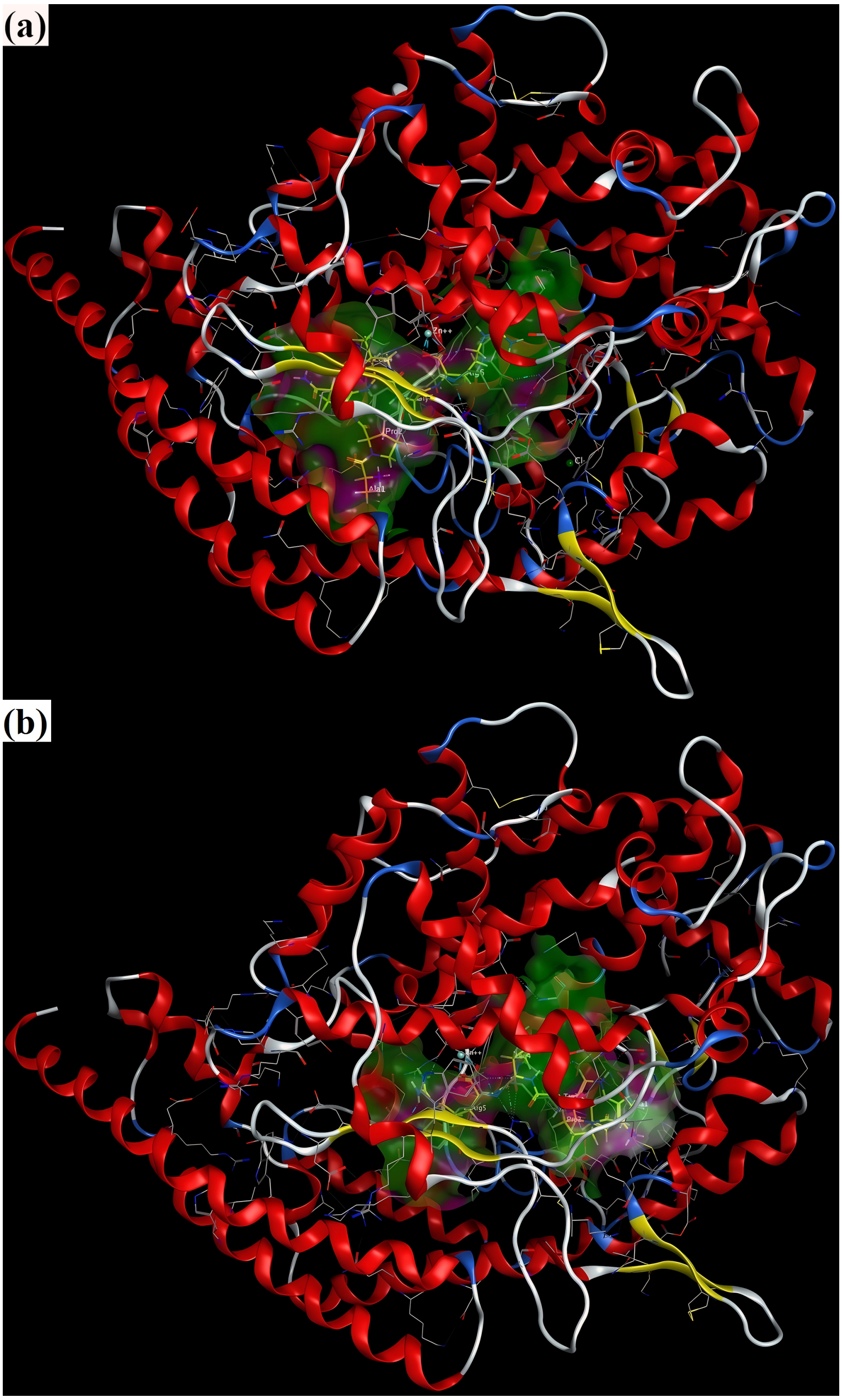

Figure S3. Molecular docking for APQLGR and APWLR with ACE (PDB: 1O86). (**a**) 3D model of APQLGR-ACE (The yellow compound represents APQLGR), (**b**) 3D model of APWLR-ACE (The yellow compound represents APWLR).
