# Supplementary material for: Preparation, Separation, and Identification of Low-Bitter ACE-Inhibitory Peptides from Sesame (Sesamum indicum L.) Protein
Source: Foods. 2026 Jan 12;15(2):279. doi: 10.3390/foods15020279 (PMC12841349; doi:10.3390/foods15020279)
Supplement: Supplementary file 1 [file foods-15-00279-s001.zip › Figure S4.pdf]

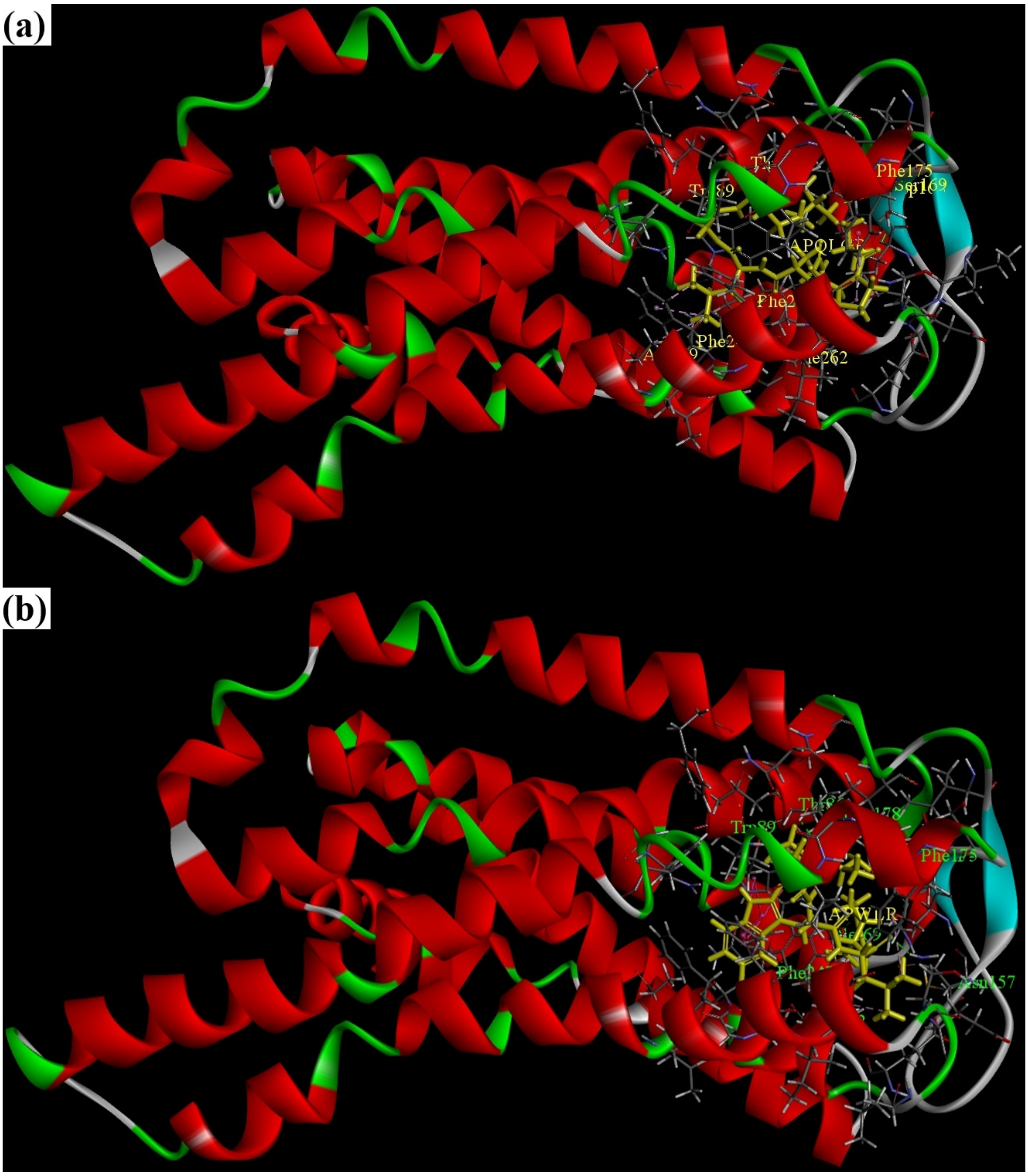

Figure S4. The 3D docking interaction of selected peptides with T2R14. (**a**) The binding structure of T2R14-APQLGR, (**b**) The binding structure of T2R14-APWLR. The yellow compounds in (**a**) and (**b**) represent APQLGR and APWLR, respectively.
