# Supplementary figures and images for: Preparation, Separation, and Identification of Low-Bitter ACE-Inhibitory Peptides from Sesame (Sesamum indicum L.) Protein
Source: Foods. 2026 Jan 12;15(2):279. doi: 10.3390/foods15020279 (PMC12841349; doi:10.3390/foods15020279)

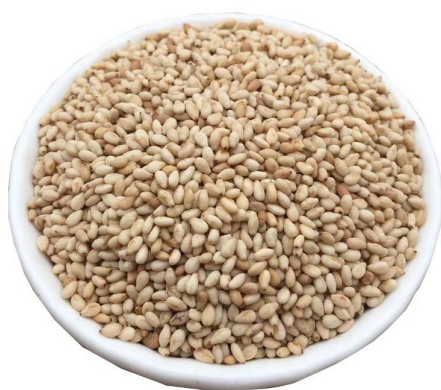

**Figure S1.** The representative picture of sesame (*Sesamum indicum* L.) seeds.

Supplement: Supplementary file 1 [file foods-15-00279-s001.zip › Figure S1.pdf]
